# Clinical Evaluation of Deep Learning for Tumor Delineation on ^18^F-FDG PET/CT of Head and Neck Cancer

**DOI:** 10.2967/jnumed.123.266574

**Published:** 2024-04

**Authors:** David G. Kovacs, Claes N. Ladefoged, Kim F. Andersen, Jane M. Brittain, Charlotte B. Christensen, Danijela Dejanovic, Naja L. Hansen, Annika Loft, Jørgen H. Petersen, Michala Reichkendler, Flemming L. Andersen, Barbara M. Fischer

**Affiliations:** 1 Department of Clinical Physiology and Nuclear Medicine, Rigshospitalet, University of Copenhagen, Copenhagen, Denmark;; 2 Department of Clinical Medicine, Faculty of Health and Medical Sciences, University of Copenhagen, Copenhagen, Denmark;; 3 Department of Applied Mathematics and Computer Science, Technical University of Denmark, Lyngby, Denmark;; 4 Department of Clinical Physiology and Nuclear Medicine, Herlev Hospital, University of Copenhagen, Copenhagen, Denmark;; 5 Section of Biostatistics, Institute of Public Health, Faculty of Health Sciences, University of Copenhagen, Denmark; and; 6 PET Centre, School of Biomedical Engineering and Imaging Science, King’s College London, London, United Kingdom

**Keywords:** ^18^F**-**FDG PET/CT, head and neck cancer, tumor volume delineation, imaging biomarkers, deep learning

## Abstract

Artificial intelligence (AI) may decrease ^18^F**-**FDG PET/CT–based gross tumor volume (GTV) delineation variability and automate tumor-volume–derived image biomarker extraction. Hence, we aimed to identify and evaluate promising state-of-the-art deep learning methods for head and neck cancer (HNC) PET GTV delineation. **Methods:** We trained and evaluated deep learning methods using retrospectively included scans of HNC patients referred for radiotherapy between January 2014 and December 2019 (ISRCTN16907234). We used 3 test datasets: an internal set to compare methods, another internal set to compare AI-to-expert variability and expert interobserver variability (IOV), and an external set to compare internal and external AI-to-expert variability. Expert PET GTVs were used as the reference standard. Our benchmark IOV was measured using the PET GTV of 6 experts. The primary outcome was the Dice similarity coefficient (DSC). ANOVA was used to compare methods, a paired *t* test was used to compare AI-to-expert variability and expert IOV, an unpaired *t* test was used to compare internal and external AI-to-expert variability, and post hoc Bland–Altman analysis was used to evaluate biomarker agreement. **Results:** In total, 1,220 ^18^F**-**FDG PET/CT scans of 1,190 patients (mean age ± SD, 63 ± 10 y; 858 men) were included, and 5 deep learning methods were trained using 5-fold cross-validation (*n* = 805). The nnU-Net method achieved the highest similarity (DSC, 0.80 [95% CI, 0.77–0.86]; *n* = 196). We found no evidence of a difference between expert IOV and AI-to-expert variability (DSC, 0.78 for AI vs. 0.82 for experts; mean difference of 0.04 [95% CI, −0.01 to 0.09]; *P* = 0.12; *n* = 64). We found no evidence of a difference between the internal and external AI-to-expert variability (DSC, 0.80 internally vs. 0.81 externally; mean difference of 0.004 [95% CI, −0.05 to 0.04]; *P* = 0.87; *n* = 125). PET GTV–derived biomarkers of AI were in good agreement with experts. **Conclusion:** Deep learning can be used to automate ^18^F**-**FDG PET/CT tumor-volume–derived imaging biomarkers, and the deep-learning–based volumes have the potential to assist clinical tumor volume delineation in radiation oncology.

## Visual Abstract

**Figure:**
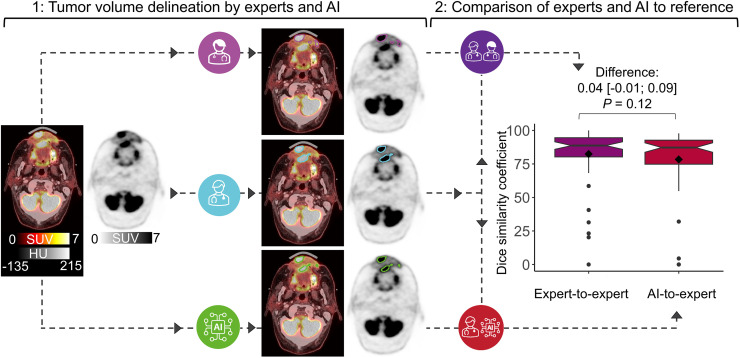

PET/CT with ^18^F**-**FDG is integral to the oncologic evaluation of nodal involvement, identification of distant metastases, radiotherapy planning, response assessment, and patient follow-up (1–4). This applies to several types of cancer, including head and neck cancer (HNC). HNC was the seventh most common cancer worldwide in 2018, with 890,000 new cases and 450,000 deaths (5). Modern chemo-, immuno-, and high-precision radiotherapy have increased survival; however, these treatments depend on advanced image analysis, including tumor delineation by expert specialists on functional and anatomic images (6).

Delineation of ^18^F**-**FDG PET/CT–based gross tumor volume (GTV) to guide radiotherapy involves distinguishing healthy from pathologic metabolic activity with high accuracy. This task is complex in assessing nodal involvement and distinguishing healthy metabolic activity in the proximity of malignant tissue. Furthermore, the anatomic closeness between organs at risk, lymph nodes, and malignant tissues can make HNC PET GTV delineation particularly challenging. Here, a high-quality automated tumor delineation method could lead to more consistent and repeatable image evaluation. Additionally, treatment delay in radiotherapy leads to a decreased chance of tumor control and an increased risk of metastases. In this context, the time savings made possible by automated contouring in ^18^F**-**FDG PET/CT–guided radiotherapy planning holds a potentially tangible clinical impact (7).

Artificial intelligence (AI) deep learning methods are currently the state of the art for semantic segmentation on medical images (8). Although lack of clinical evaluation has hampered AI implementation in practice, there is good evidence that predefined deep learning methods can solve advanced, previously unseen problems (9–11). Considering the success of predefined deep learning methods with novel problems, we hypothesized that these algorithms could delineate PET GTV to a standard comparable to our current clinical methods.

In this study, we identified promising, reproducible, predefined AI deep learning methods in a systematic literature review and trained them on a large clinical dataset. We investigated how the methods compared with clinical PET GTVs. We evaluated AI on internal and external test scans, using expert-delineated PET GTVs as the reference. Finally, we investigated whether PET GTV–derived metabolic biomarkers were reliable. We aimed to identify, train, and evaluate promising state-of-the-art deep learning methods for HNC PET GTV delineation and PET GTV–derived biomarker extraction.

## MATERIALS AND METHODS

This retrospective clinical evaluation was approved by the Danish Patient Safety Authority (approval 31-1521-340, reference no. SMMO) and the Danish Data Protection Agency (approval P-2020-427).

Observer evaluations were conducted from January to October 2022, the analysis was performed from October 2022 to March 2023, and the statistical analysis plan was published in September 2022 before looking at any data at the registry (www.isrctn.com/: ISRCTN16907234). After internal results became available, the decision was made to test deep learning on an external dataset.

A full version of the Materials and Methods section is available as Supplemental Appendix A (12–15). Supplemental Appendix B and Supplemental Table 1 list image acquisition and preprocessing details. Hardware and software specifications are listed in Supplemental Table 2 (supplemental materials are available at http://jnm.snmjournals.org).

### Study Design and Patient Population

**FIGURE 1.**
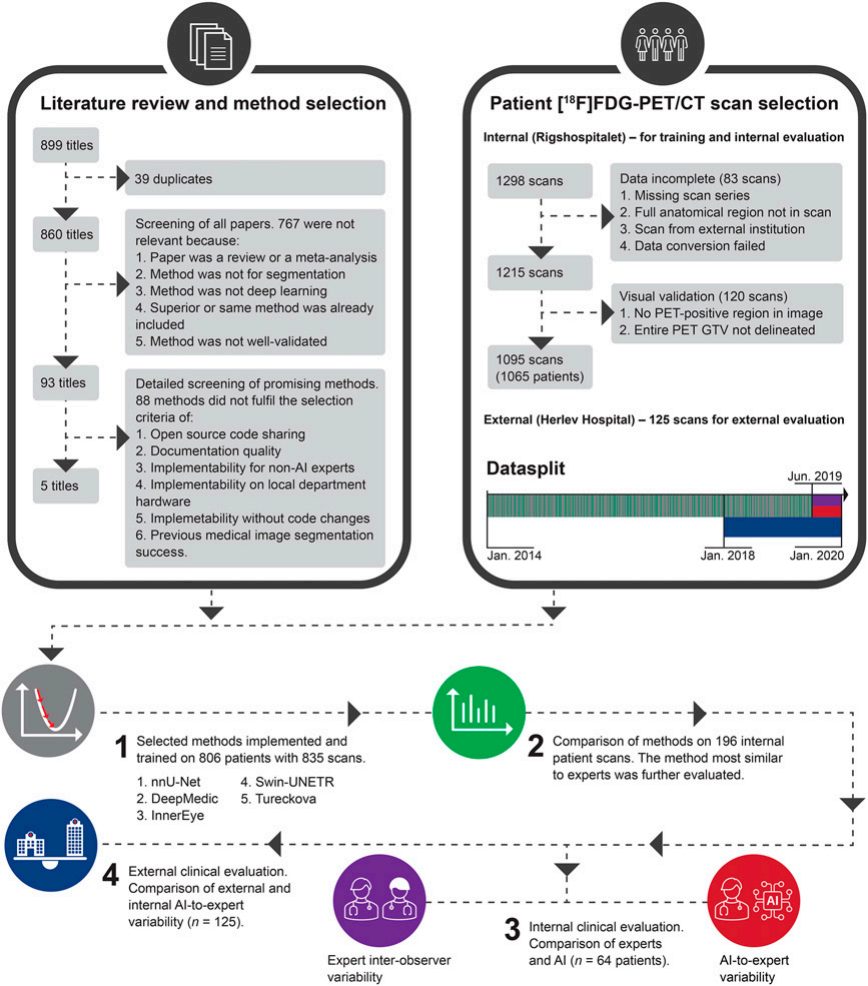
Summary of study. Causes of exclusion were data incompleteness and failure to pass visual validation. No external scans were excluded to these criteria. Model training used 805 patients (835 scans). Each scan represented unique patient in steps 2, 3, and 4.

Scans were included of patients with HNC referred for ^18^F**-**FDG PET/CT–guided radiotherapy at our institution (internal) between 2014 and 2019 and an external institution between 2018 and 2019. The reasons for exclusion were incomplete data or failure to pass visual validation (Fig. 1). No patients were below 18 y of age. The selected scans were divided into training, validation, and test subsets. Three test sets, including an external one, were created with sample sizes based on power considerations.

The institutional review boards at the involved hospitals approved this retrospective study, and the requirement to obtain informed consent was waived.

### Clinical Evaluation and Reference Delineations

The clinical PET GTVs used for radiotherapy were applied for model training and methods comparison. In our clinical routine, the PET GTV delineated by nuclear medicine specialists is sent to a radiation oncologist or radiologist, who uses the volume to guide the final GTV delineation. Hence, for clinical evaluation, PET GTV was independently delineated on the test sets for this study by 7 nuclear medicine specialists: 6 internally and 1 externally (board-certified specialists with 3.5 to >15 y of experience). The PET GTV region was delineated on the PET image with an optionally underlying CT image. A visually adapted isocontour without a fixed threshold was used to fit the steepest gradient between the ^18^F-FDG–avid malignant region and the surrounding tissue, excluding areas with nonmalignant uptake.

**FIGURE 2.**
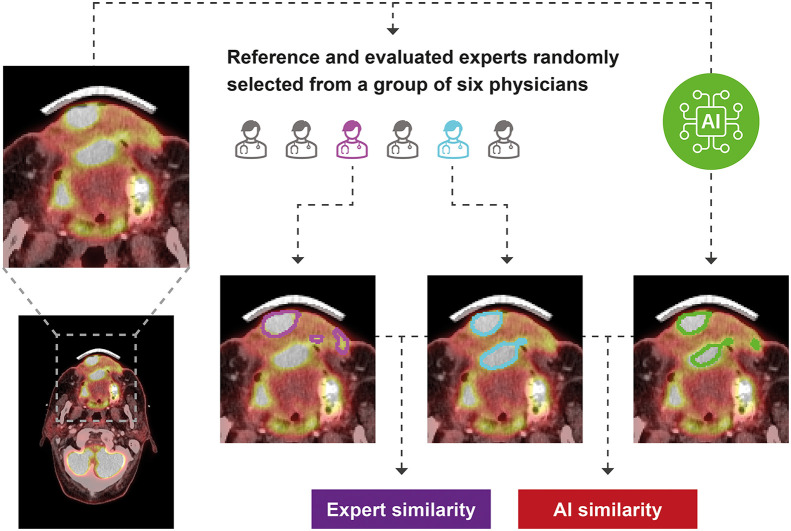
Study design comparing expert IOV and AI-to-expert variability. Delineations are exemplified on random patient’s axial ^18^F-FDG PET/CT intravenous contrast scan slice.

Interobserver variability (IOV) was evaluated by 2 randomly selected experts from a group of 6 delineating the PET GTV on each scan (Fig. 2). The randomization was blocked; each expert therefore contributed equally. Scans were uploaded to clinical systems twice under different anonymous identities to ensure that experts could not review previous PET GTVs. To resemble clinical practice, where it is random which of our experts delineate each scan, 1 of the 2 experts was randomly selected as the reference. The patients were anonymized to mask the experts. Hence, the internal experts could not look up contextual patient journal information. Instead, they were provided with a brief text containing the causes of the referral. The external expert had access to the same information as in clinical routine, except for the PET GTV used for treatment.

### Statistical Analysis

We selected the Dice similarity coefficient (DSC) expressing the degree of overlap (0, no overlap; 1, complete overlap) between 2 volumes because it is the most widely used metric in the deep learning literature, allowing for comparison to other studies. The Hausdorff distance, F1 score, PET GTV–derived tumor volume (cm^3^), SUV_mean_, and SUV_max_ were secondary outcomes. We used a significance level of 0.05 in all statistical testing.

The sample sizes were based on power calculations in R using a power of 0.8, a significance level of 0.05, and an expected SD of 0.14, based on measurements published by Gudi et al. (14). In the ANOVA, we aimed to show a DSC difference of at least 0.025. The *t* test power calculations used a 2-sided alternative hypothesis. We aimed to show a DSC difference of at least 0.05. The intended sample sizes were 196 to compare methods’ AI-to-expert variability using 1-way ANOVA, 64 to compare AI-to-expert variability and expert IOV using a paired *t* test, and 125 to compare internal to external AI-to-expert variability using an unpaired *t* test (the latter comparing 2 different patient groups). A significance level of 0.05 was used in all statistical evaluations. Post hoc Bland–Altman analysis was used to evaluate biomarker agreement. The tumor volumes were modeled as a sphere, of which we calculated the radius to achieve interpretable yet normally distributed differences. We selected and described cases in which AI or experts failed.

## RESULTS

### Patient Characteristics

We found 1,298 ^18^F**-**FDG PET/CT scans for radiotherapy planning from servers at Rigshospitalet (the internal institution). Of these, 83 were excluded because of incomplete data, and 120 failed to pass visual validation (Fig. 1). This left 1,095 scans from 1,065 patients. All 125 scans from the external institution (Herlev Hospital) were included, leading to a total of 1,190 patients studied (mean age ± SD, 63 ± 10 y; 858 men; Table 1).

**TABLE 1.** Summary of Patient Demographics and Key Clinical Characteristics in Each Dataset

Characteristic	Training and validation	Method comparison	Internal clinical evaluation	External clinical evaluation
Number of patients	805	196	64	125
Age (y)	62.6 ± 10.5	62.8 ± 9.3	65.6 ± 10.0	64.2 ± 8.6
Weight (kg)	75.0 ± 18.1	76.3 ± 18.7	71.8 ± 16.9	74.8 ± 18.6
Dose (MBq)	300.4 ± 72.1	306.8 ± 72.9	283.1 ± 67.6	289.1 ± 61.5
Injection-to-scan time (h)	1.1 ± 2	1.1 ± 3	1.1 ± 1	1.1 ± 1
Sex	584 (73%) men	141 (72%) men	42 (66%) men	90 (72%) men
Oropharynx	301/805 (37%)	78/196 (40%)	23/64 (40%)	76/125 (61%)
Larynx	123/805 (15%)	30/196 (15%)	9/64 (15%)	7/125 (6%)
Cavum oris	87/805 (11%)	22/196 (11%)	7/64 (11%)	8/125 (6%)
Hypopharynx	84/805 (10%)	21/196 (11%)	5/64 (8%)	22/125 (18%)
Rhinopharynx	50/805 (6%)	12/196 (6%)	6/64 (9%)	5/125 (4%)
Vestibulum nasi or sinus paranasalis	33/805 (4%)	6/196 (3%)	3/64 (2%)	1/125 (1%)
Unknown primary with lymph nodes	18/805 (2%)	3/196 (2%)	0/64 (0%)	6/125 (5%)
Salivary gland tumor	10/805 (1%)	0/196 (0%)	0/64 (0%)	0/125 (0%)
Unspecified	99/805 (12%)	24/196 (12%)	12/64 (19%)	0/125 (0%)

Qualitative data are number and percentage; continuous data are mean and SD (total *n* = 1,190). There was no difference in age between men and women in any of 4 datasets (all *P* > 0.05). Mean age was higher in internal clinical evaluation test set than in training data (*P* = 0.01). At same time, there was no evidence of age differences from training data in 2 other test sets (*P* = 0.74 for method comparison test set and *P* = 0.10 for external test set).

We allocated 4 patient cohorts for this study: 1 cohort for training/validation (*n* = 835) trained with 5-fold cross-validation and 3 cohorts for testing (Table 2). Test 1 (*n* = 196) was used for model evaluation and selection of the optimal model. The model achieving the highest performance was used in subsequent tests. Test 2 (*n* = 64) was used to compare this model with human experts. All subjects in the test 2 cohort were scanned at a later date than the training and test 1 cohort (Table 2) to ensure no model selection bias based on subjects. Finally, in test 3 (*n* = 125), an external evaluation was performed with externally acquired data.

**TABLE 2.** Summary of Datasets for Model Training, Validation, and Independent Testing

Set	Start	End	Source	*n*	Purpose
Training by cross-validation					
Training folds (80%)	2014 January	2019 June	Internal	668	Train model
Validation fold (20%)	2014 January	2019 June	Internal	167	Validate model
Test					
1	2014 January	2019 June	Internal	196	Compare models performance
2	2019 July	2019 December	Internal	64	Compare models with experts’ IOV
3	2018 January	2019 December	External	125	Compare internal with external model performance

### Description of Included Methods

Of 860 unique results, 767 were excluded because of lack of direct relevance to this study (metaanalyses, task not segmentation, method not deep learning, superior method included, or method not well validated). Of the remaining 93 titles, 88 were excluded because of lack of open-source, documentation, and implementability (further details and search key words in Supplemental Appendix A and Fig. 1). All methods were trained with a 2-channel PET and CT input against PET GTV as the output.

Five methods met our inclusion criteria (Fig. 1): nnU-Net, DeepMedic, InnerEye, Swin-UNETR, and Tureckova. nnU-Net (version 1) (16) is based on the U-Net (17), characterized by a U-shaped architecture consisting of convolutional and pooling layers mirrored to form the output. nnU-Net is designed to deal with dataset diversity by fully standardizing and automating the pre- and postprocessing design decisions based on training data features. DeepMedic (18) uses a dual pathway of convolutional layers that simultaneously process the image at normal and low resolution to incorporate local and contextual information. Subsequently, it uses a 3-dimensional fully connected conditional random field model to remove false positives. InnerEye (19) features a HeadAndNeckBase class used in a previous publication about HNC tumor segmentation. This was used in our implementation. The method uses a 3-dimensional U-Net with strided convolutions instead of max-pooling operations, nonlinear activation of upsampled tensors, residual connections, and dilated convolution kernels in the encoder to preserve more contextual information. Swin-UNETR (20) attempts to solve the challenge of modeling long-range information using shifting window transformers that compute self-attention in an efficient shifted window partitioning scheme. It utilizes the U-shaped architecture with a shifting window transformer as the encoder and connects it to a convolutional neural network–based decoder at different resolutions via skip connections. Finally, Tureckova (21) is an extension to nnU-Net featuring a V-Net architecture with attention gates designed to help the network focus on a desired scan area by learning to focus on a subset of target structures.

### Comparison of AI Methods

**FIGURE 3.**
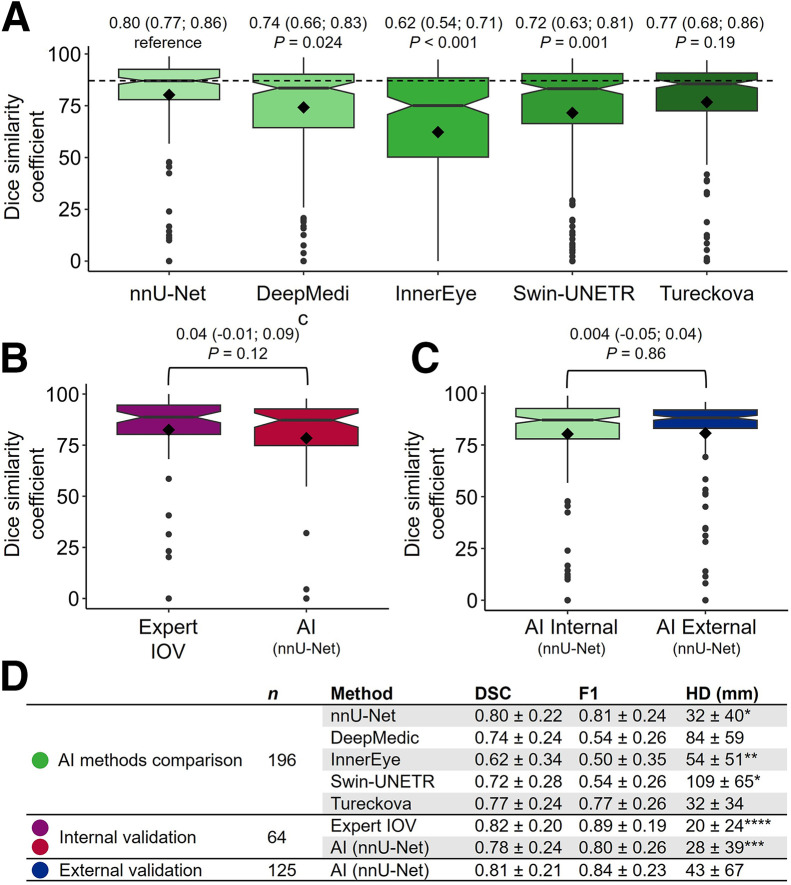
(A) Comparison of 5 implemented methods trained on 196 patient scans based on DSC. Values above boxes are mean followed by 95% CI in parentheses, with *P* values below. nnU-Net achieved highest DSC and was further analyzed (denoted AI). (B) Paired comparison of AI-to-expert variability and expert IOV on 64 independent internal test scans. (C) Comparison of AI-to-expert variability on 196 internal (same as nnU-Net in A) and 125 external patients. All 3 comparisons used expert-delineated tumor volumes as reference. Values above boxes in B and C are mean difference followed by 95% CI in parentheses, with *P* values below. Rhombus shape indicates mean value, and central line represents median. Boxes enclose interquartile range. Whiskers extend to most significant measurement no further than 1.5 × interquartile range from hinge. Data beyond whiskers are plotted individually. Notch roughly represents 95% CI around median. (D) DSC, F1 score (F1), and Hausdorff distance (HD) summary statistics in mean ± SD. Hausdorff distance is undefined when expert or AI includes no volume. Hence, numbers marked with *, **, ***, and **** were based on *n* = 195, 186, 61, and 63, respectively.

**FIGURE 4.**
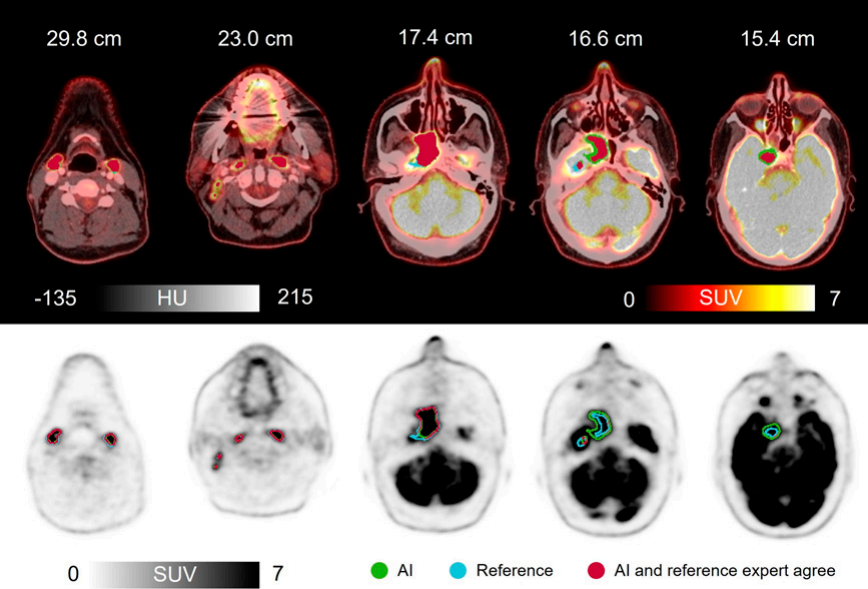
Clinical scan delineated by expert (reference) and by AI, along with AI-to-expert agreement (DSC, 0.92). Shown are axial images of 50-y-old man with HNC of rhinopharynx. ^18^F**-**FDG PET/CT with intravenous contrast agent showed greatly increased activity corresponding to large tumor process in right rhinopharynx, crossing midline and growing frontally into cavum nasi on right, intracranially on right, medially in fossa media, and along dura laterally. In addition, multiple lymph nodes in neck had greatly increased activity bilaterally. AI correctly avoided including physiologically active areas such as saliva, metal artifact–induced activity, nose tip, brain, and optic nerve. HU = Hounsfield units.

nnU-Net achieved the highest mean DSC (Fig. 3A, clinical example in Fig. 4). The DSCs of DeepMedic, InnerEye, and Swin-UNETR were lower (all *P* ≤ 0.05, exact values in Fig. 3), whereas we found no evidence of a difference from Tureckova (*P* = 0.19). Complete delineation disagreement (DSC, 0) between clinical and AI-based delineations occurred in 4 of 196 patients (2%) for DeepMedic; 6 of 196 patients (3%) for nnU-Net, Swin-UNETR, and Tureckova; and 35 of 196 patients (18%) for InnerEye. nnU-Net achieved a higher F1 score than Tureckova. Hence, nnU-Net, hereafter referred to as AI in this Results section, was further evaluated.

### Internal Clinical Evaluation

The internal clinical evaluation showed no evidence of differences between AI-to-expert variability (DSC, 0.78) and expert IOV (DSC, 0.82, a mean difference of 0.04 [95% CI, −0.01 to 0.09]; *P* = 0.12; Fig. 3B). In 1 patient, AI included no volume, whereas the experts did. The reference expert included no volume in 2 cases, whereas the other expert and AI did.

**FIGURE 5.**
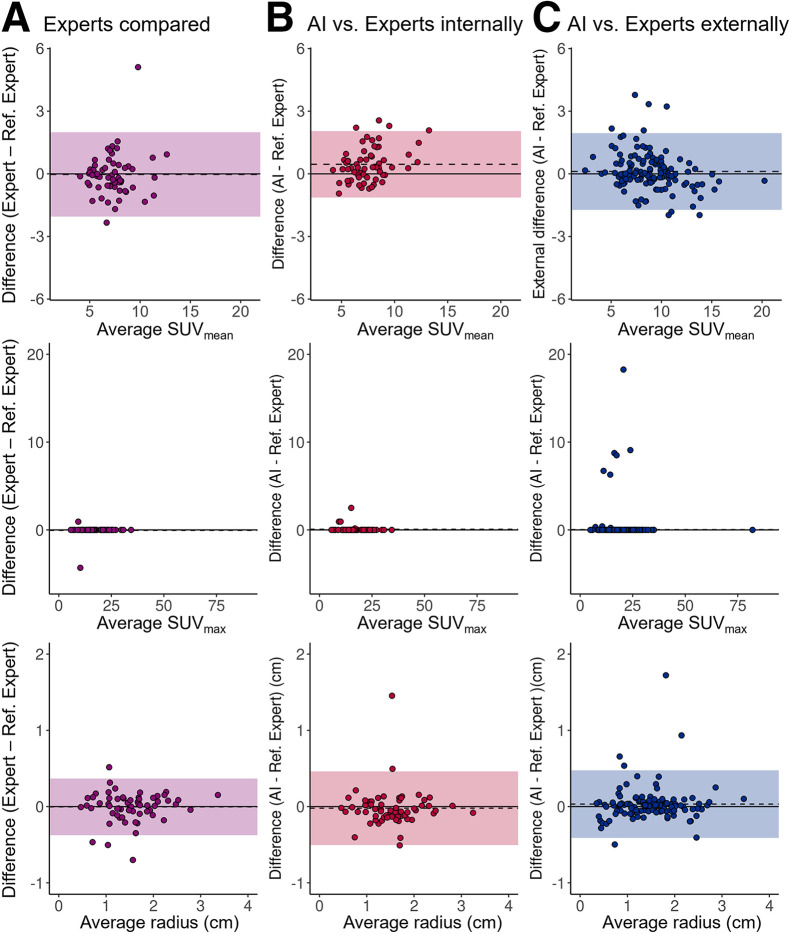
Tumor-volume–derived biomarkers based on AI delineation in good agreement with experts. Shown are Bland–Altman plots for agreement between AI and expert PET GTV–derived biomarkers. Shaded regions and dashed lines represent LoA and mean bias, respectively. Limits of agreement for SUV_max_ were not included because of violation of normality assumption. To improve visualization, single outlier with extreme SUV_max_ of 151 in B was excluded from plot.

AI and expert PET GTV–based biomarker agreement was acceptable (Figs. 5A and 5B). The 3 scans with no PET GTV detected were excluded, leaving 61 patients for analysis. For SUV_mean_, AI showed narrower limits of agreement (LoAs) than experts compared (lower to upper, −1.1 to 2.1 for AI and −2.1 to 2.0 for experts). We found no evidence of bias between experts (*P* = 0.83), whereas AI overestimated values by 0.5 (95% CI, 0.2–0.7) (*P* ≤ 0.001). Tumor radius LoAs of AI were broader than experts’ (lower to upper, −0.5 to 0.5 cm for AI and −0.4 to 0.4 cm for experts), and we observed no evidence of biases (0.004 cm [95% CI, −0.05 to 0.05 cm; *P* = 0.88] for the experts and 0.02 cm [95% CI, −0.08 to 0.04 cm; *P* = 0.51] for AI). SUV_max_ was not analyzed statistically because of violation of the normality assumption; however, AI and experts found the same value in all except 3 patients (Supplemental Fig. 2), whereas experts found the same value in all but 2 patients.

### External Clinical Evaluation

The external clinical evaluation showed no evidence of a difference between internal and external AI-to-expert variability (mean DSC difference of 0.004 [95% CI, −0.05 to 0.04; *P* = 0.86], Fig. 3C). Both the expert and AI identified PET GTV in all patients.

External biomarker agreement between AI and experts was acceptable (Fig. 5C). For SUV_mean_, the LoA was narrower externally than that of internal experts (lower to upper, −1.7 to 2.0 externally and −2.1 to 2.0 internally), whereas no bias was detected (0.1 [95% CI, −0.1 to 0.3]; *P* = 0.18). For the tumor volume radius, Lower and upper LoAs for the PET GTV were −0.4 to 0.5 cm externally and −0.5 to 0.5 cm internally, and there was no evidence of bias (0.03 cm [95% CI, −0.007 to 0.07 cm]; *P* = 0.10). Because of violation of the normality assumption, SUV_max_ was not analyzed statistically; however, experts and AI found the same value in 116 of 125 patients.

### Failure Analysis

We identified 11 patients for whom either AI or experts failed (Supplemental Fig. 1). The main causes of delineation failure for AI and experts were postsurgical inflammation (4 patients) and lymph node inclusion disagreements (4 patients). In addition, rare situations not represented in the training scans could have led AI to fail (2 patients): one in which physicians included a tonsil with borderline activity and one in which the patient was lying in a nonstandard position in the scanner. Finally, for a single patient, the reference physician incorrectly included a region in the patient’s orbita. Six of the 11 test scans were postsurgical (55%). The test set featured 10 of 64 postsurgical patients (16%). In postsurgical patients, expert DSC dropped from 0.82 to 0.58. AI DSC dropped from 0.78 to 0.46. Excluding postsurgical patients and repeating the paired *t* test to compare AI-to-expert variability and expert IOV, the lack of a significant difference between the AI-to-expert variability and expert IOV remained (DSC, 0.87 for AI-to-expert vs. 0.84 for expert IOV, a difference of 0.03 [95% CI, −0.02 to 0.07]; *P* = 0.24).

## DISCUSSION

This study was conducted to identify and evaluate state-of-the-art deep learning for delineating PET GTV in HNC and for PET GTV–derived biomarker extraction. We demonstrated that deep learning could delineate volumes similar to clinical quality (AI-to-expert DSC, 0.78, and IOV of 6 experts’ DSC, 0.82; *P* = 0.12) and that resulting PET GTV–derived biomarkers were reliable (tumor volume radius upper and lower limits of agreement, −0.5 to 0.5 cm for AI and −0.4 to 0.4 cm for experts). We suspect most clinics will find these limits of agreement acceptable for clinical use. Further, our results warrant further investigation into how deep learning could reduce clinical tumor delineation variability.

During our survey of deep learning methods, we encountered 93 promising titles. Of these, 88 lacked shared code, proper documentation, or accessible implementability. nnU-net achieved the highest level of similarity to our experts. This method has had a significant impact in recent years, winning challenges such as the Brain Tumor Image Segmentation Benchmark and the Medical Segmentation Decathlon (11,22). Notably, since 2022, the leading method of the latter challenge has been the Swin-UNETR method, which we expected would be superior to nnU-Net. However, this was not the case. This difference may imply that nnU-Net is the more robust method when pretraining data are unavailable; however, the results may be specific to our setting. Compared with other models, nnU-Net has the benefit of flexibility enabled by integrated self-configuring pre- and postprocessing, enabling reliable results for various tasks. Conversely, the main weakness of nnU-Net is its computational demand. Like other deep learning models, nnU-Net lacks inherent interpretability and depends on high data quality. Although the Head and Neck Tumor Segmentation Challenge (HECKTOR) is not directly comparable because of segmentation task differences, it resembles our work. Here, the nnU-Net achieved a similarity in our study as observed in the latest results of HECKTOR (DSC, 0.8). Finally, although we have not explicitly tested model performance using CT or PET alone, it has previously been shown that a multimodal PET/CT input is superior to either modality used alone (23).

If a method has limited precision, even a perfect new method will not agree with it (24). Hence, the AI-to-expert similarity can exceed the expert IOV only by random effect, making IOV measurements important to understand the level of similarity to experts that AI can theoretically achieve. Although some authors provide IOV and find similar results to this work (25), such data are often unavailable. With a DSC of 0.82, our experts achieved a higher similarity than other groups (DSC, 0.61–0.69) (14,26). Notably, we used the delineation of a random expert as the reference for each patient, which we consider a considerable strength because the resulting variability represents what patients are exposed to in practice (for instance, consensus delineations are not used in clinical practice).

We do not know of others evaluating deep learning–based biomarker extraction from PET/CT scans of HNC. However, there is evidence that PET/CT biomarkers can be extracted for ^68^Ga-PSMA PET/CT scans of prostate cancer (total lesion volume and uptake) and ^18^F-FDG PET/CT scans of lymphoma (total metabolic tumor volume) (27,28). These results support the indication of this work that AI can safely be used for PET/CT biomarker extraction.

Our failure analysis showed that expert IOV and AI-to-expert variability increased in postsurgical scans. In addition, AI tended to include more lymph nodes than physicians did. Concerning postsurgical patient scans, our observation is consistent with the literature (29). Furthermore, the observation matches our clinical experience: scans taken after surgery complicate the classification of ^18^F-FDG–avid regions. Concerning lymph nodes, the clinical decision of inclusion relies on the optimal balance between including too much or too little tissue. Hence, the final delineation result depends on a clinical risk assessment of contextual information. Considering this information in a deep learning model requires methodologic developments before being tested in a clinical context.

Our study had limitations. We confined the inclusion of methods to those reproducible in our context, which could lead to the exclusion of leading methods. For example, the 2022 HECKTOR winner did not meet our method selection criteria (30). Furthermore, because of masking, the internal experts did not have access to routine contextual information, a necessary precaution to avoid the potential bias from seeing tumor-volume delineations of other experts. Finally, although several scanners were represented, scans were performed mostly on the same model (Siemens Biograph 64), which could limit generalization.

Our findings indicate that AI-based PET GTV delineations are on a par with human delineations, bearing significant implications for clinical practice. First, it could reduce delineation time for nuclear medicine physicians, radiologists, and radiation oncologists. Second, AI’s consistency could reduce physician IOV. Consequently, this points to potential improvements in the speed and consistency of treatment planning and increased biomarker stability.

## CONCLUSION

Deep learning can be used for automated PET GTV–derived biomarker extraction and large imaging biomarker studies. Furthermore, deep learning can delineate PET GTVs similar to clinical volumes, holding potential for radiotherapy planning.

## DISCLOSURE

David Kovacs received research funding from Brødrene Hartmanns Fond. Barbara Fischer received research funding from Aage and Johanne Louis-Hansen Fonden and has received support from Siemens Healthineers to attend meetings. No other potential conflict of interest relevant to this article was reported.

## Notes

KEY POINTS **QUESTION:** Can AI deep learning automate ^18^F-FDG PET/CT HNC tumor volume delineation and image biomarker extraction? **PERTINENT FINDINGS:** We retrospectively developed 5 deep learning models using 5-fold cross-validation and compared them using the DSC as the primary outcome. The AI-to-expert similarity (DSC, 0.78) was not significantly different from expert-to-expert similarity (DSC, 0.82), and when evaluated in Bland–Altman analysis, the AI imaging biomarker limits of agreement were in the same range as the uncertainty between experts. **IMPLICATIONS FOR PATIENT CARE:** Deep learning can automate ^18^F-FDG PET/CT biomarker extraction in HNC. It can potentially assist tumor delineation in radiotherapy planning.
